# Isolation and screening of l-asparaginase free of glutaminase and urease from fungal sp.

**DOI:** 10.1007/s13205-016-0544-1

**Published:** 2016-11-12

**Authors:** Kruthi Doriya, Devarai Santhosh Kumar

**Affiliations:** Department of Chemical Engineering, Industrial Bioprocess and Bioprospecting Laboratory, Indian Institute of Technology Hyderabad, Room No: 530, Kandi Campus, Kandi, Medak Dist, Hyderabad, Telangana State 502285 India

**Keywords:** l-Asparaginase, l-Glutaminase, Urease, Glutaminase-free l-asparaginase, Urease and glutaminase-free l-asparaginase

## Abstract

l-Asparaginase is a chemotherapeutic drug used in the treatment of acute lymphoblastic leukaemia (ALL), a malignant disorder in children. l-Asparaginase helps in removing acrylamide found in fried and baked foods that is carcinogenic in nature. l-Asparaginase is present in plants, animals and microbes. Various microorganisms such as bacteria, yeast and fungi are generally used for the production of l-asparaginase as it is difficult to obtain the same from plants and animals. l-Asparaginase from bacteria causes anaphylaxis and other abnormal sensitive reactions due to low specificity to asparagine. Toxicity and repression caused by bacterial l-asparaginase shifted focus to eukaryotic microorganisms such as fungi to improve the efficacy of l-asparaginase. Clinically available l-asparaginase has glutaminase and urease that may lead to side effects during treatment of ALL. Current work tested 45 fungal strains isolated from soil and agricultural residues. Isolated fungi were tested using conventional plate assay method with two indicator dyes, phenol red and bromothymol blue (BTB), and results were compared. l-Asparaginase activity was measured by cultivating in modified Czapek–Dox medium. Four strains have shown positive result for l-asparaginase production with no urease or glutaminase activity, among these C_7_ has high enzyme index of 1.57 and l-asparaginase activity of 33.59 U/mL. l-Asparaginase production by C_7_ was higher with glucose as carbon source and asparagine as nitrogen source. This is the first report focussing on fungi that can synthesize l-asparaginase of the desired specificity. Since the clinical toxicity of l-asparaginase is attributed to glutaminase and urease activity, available evidence indicates variants negative for glutaminase and urease would provide higher therapeutic index than variants positive for glutaminase and urease.

## Introduction

l-Asparaginase is an amidohydrolase that catalyses l-asparagine to l-aspartate and ammonia. l-Asparaginase is found to have tumour inhibitory properties. It is mainly used in the treatment of acute lymphoblastic leukaemia (ALL). Normal cells can synthesize l-asparagine with the help of asparagine synthetase, whereas certain sensitive malignant cells cannot synthesize it by itself and require an external source of l-asparagine for growth. During the treatment of ALL with l-asparaginase, all the circulating asparagine in the body of the patient get hydrolysed to aspartic acid and ammonia preventing the absorption of asparagine by tumour cells thereby depriving the tumour cells of their extracellular source of l-asparagine (Broome 1961). L-Asparaginase is commonly used as a combination chemotherapy drug for the treatment of acute lymphoblastic leukaemia (ALL) in adults and children and non-Hodgkin’s lymphoma in children (Mashburn and Wriston 1964). l-Asparaginase also reduces acrylamide formation in food by selectively hydrolysing asparagine to aspartic acid and ammonia without affecting other amino acids, retaining food quality. Application of l-asparaginase enzyme (2 U/g) successfully reduced acrylamide content by 90 % in potato products that have high asparagine content (Friedman 2003; Ciesarová et al. 2006).

l-Asparaginase is widely present in plants, animals and microbes but not in humans. Microbes are a better source for the production of enzyme as they are easy to cultivate and manipulate (Kumar and Sobha 2012). Clinically three asparaginase formulations are available, two from bacterial sources *Escherichia coli* (*E. coli* asparaginase) and *Erwinia chrysanthemi* (*Erwinia* asparaginase) and PEGylated form of *E. coli* asparaginase. L-Asparaginase therapy has side effects such as anaphylaxis, coagulation abnormality, thrombosis, liver dysfunction, pancreatitis, hyperglycaemia and cerebral dysfunction, etc.These side effects are either due to the production of anti-asparaginase antibody in the body or l-glutaminase activity of l-asparaginase enzyme (Haskell et al. 1969; Mahajan et al. 2012). Toxicity of l-asparaginase is mainly due to the fact that the enzyme preparations are amidohydrolase, not l-asparaginase. Clinically available l-asparaginase shows notable hydrolysis of l-glutamine and d-asparagine, signifying multiple enzyme activities contaminating enzyme preparation and difficult to eliminate other enzymes (Campbell and Mashburn 1969). Notwithstanding numerous studies on bacterial l-asparaginase, treatment with it sometimes results in hypersensitive reactions such as anaphylactic shock. l-Asparaginase isolated from filamentous fungi, *Aspergillus terreus* showed a greater carcinostatic effect on static tumour (De-Angeli et al. 1970). Similar effect was observed when l-asparaginase from deuteromycetes *Fusarium tricinctum* was purified which regressed lymphosarcoma in mice (Scheetz et al. 1971). Later purified extracellular l-asparaginase from *A. terreus* was conjugated with polyethylene glycol and it did not indicate any glutaminase activity (Loureiro 2012). Sarquis et al. examined *Aspergillus tamari* and *A. terreus* for l-asparaginase production and found that asparaginase activity is reduced in the presence of urea and glutamine (Sarquis et al. 2004). On the other hand, Bano and Sivaramakrishnan discovered that purified l-asparaginase from green chillies showed presence of glutaminase and urease. Further studies revealed that urease is present in *E. coli* enzyme preparation, which may result in toxic effects by hydrolysis of blood urea (Bano and Sivaramakrishnan 1980). Manna et al. produced and purified l-asparaginase from *Pseudomonas stutzeri* MB-405 which showed high specificity towards asparagine but did not hydrolyze glutamine, also asparaginase activity was lacking at 2 M urea (Manna et al. 1995). Therefore, current study is an effort to isolate fungi that can produce asparaginase free of glutaminase and urease. This process involves isolation of fungi from soil and agricultural residue for extracellular synthesis of l-asparaginase.

## Materials and methods

l-Asparagine was procured from Sigma-Aldrich, India. Other chemicals used were of analytical grade. *Aspergillus terreus* MTCC 1782 was obtained from Microbial Type Culture Collection Centre and Gene Bank, Institute of Microbial Technology, Chandigarh, India.

### Isolation of fungi from collected samples

Soil samples were collected from different locations of Vizag, Kanyakumari and Kerala as mentioned in Table 1. Soil and substrate samples were collected in air-tight containers and kept at room temperature in laboratory. Fungi were isolated by serial dilution of soil and agricultural residues, and plated on modified Czapek–Dox (MCD) agar plates with l-asparagine as a sole nitrogen source and incubated at 30 °C for 96 h. Fungal strains showing change were selected and grown on potato dextrose slants.

**Table 1:** Fungal species isolated from diverse sources for the production of l-asparaginase

Source	Region	Description	No. of isolates
Soil	Kanyakumari	Soil samples were collected from different locations of sea shore	10
	Vizag	Soil samples were collected from different locations of sea shore	8
	Kerala	Soil samples were collected from Western ghats, corresponding to coordinates 9.8403°N, 77.0353°E	9
Agricultural residues	Cottonseed oil cake	Substrates were collected from local market	18
	Rice husk		
	Wheat bran		
	Red gram animal feed		

### Screening studies

#### Semi-quantitative assay for l-asparaginase producing fungi

MCD medium with composition glucose 2 g/L, l-asparagine 10 g/L, KH_2_PO_4_ 1.52 g/L, KCl 0.52 g/L, MgSO_4_·7H_2_O 0.52 g/L, FeSO_4_·7H_2_O trace, ZnSO_4_·7H_2_O trace, CuNO_3_·3H_2_O trace and agar 18 g/L was prepared (Gulati et al. 1997). About 2.5 % (w/v) stock solution of the phenol red dye was prepared and MCD medium was supplemented with 0.009 % phenol red dye. 0.04 % (w/v) of stock solution of the bromothymol blue dye was prepared and 0.007 % BTB dye was supplemented in MCD medium. Final pH of the media was adjusted to 5.5 using 1 M NaOH (Mahajan et al. 2013). Prepared media was autoclaved and poured into pre-sterilized plates. Control plates were prepared with NaNO_3_ as sole nitrogen source. MCD plates were inoculated with isolated fungi as test organism and *A. terreus* MTCC 1782 as positive test. Colony diameter and zone diameter for all the test organisms were measured and respective zone index was calculated after 72 h of incubation. Morphological observation of positive isolates was done by the method of staining and observing fungal spores using lacto phenol cotton blue staining solution.

#### Plate assay for l-glutaminase

l-Glutaminase activity of the fungal strains was detected by supplementing MCD medium with l-Gln as sole nitrogen source. Test strains were inoculated and observed for colour change from yellow to pink in case of phenol red dye and yellow to blue for BTB dye.

#### Plate assay for urease

MCD medium without nitrogen source was autoclaved and 1 % filter-sterilized urea solution was added to MCD media for detection of urease-producing fungi. Test strains were inoculated and observed for change in the colour of the medium.

### Quantitative detection of l-asparaginase assay

Quantitative determination of l-asparaginase activity was carried out using selective strains (MTCC 1782, C_3_, C_7_, W_3_ and W_5_). These strains were cultivated on potato dextrose slants at 30 °C for 96 h. From these, 1 mL of conidial suspension was inoculated into Erlenmeyer flask containing 50 mL of MCD medium with initial pH of 6.2. Flasks were incubated at 30 °C at 180 rpm for 96 h. Samples were withdrawn every 24 h to determine enzyme activity.

#### Effect of carbon and nitrogen sources

To investigate the effect of different carbon sources on l-asparaginase production, fructose, glucose, maltose, sucrose, lactose and starch were added at concentration of 0.2 %(w/v) to the MCD medium. Influence of nitrogen source on asparaginase production was obtained by substituting asparagine of MCD medium with yeast extract, peptone and sodium nitrate at a concentration of 1 % (w/v). 1 mL of C_7_ suspension (with 10 × 10^6^ cells/mL) was inoculated and flasks were incubated at 30 °C at 180 rpm for 72 h. Supernatant was used to determine asparaginase activity and protein content. The effect of inoculum volume at different levels was investigated by employing C_7_ in MCD medium.

l-Asparaginase activity is obtained by measuring the ammonia liberated using Nesslerization method by spectrophotometric analysis at 425 nm as described by Kumar et al. (2011). Enzyme assay mixture consisted of 900 µL of freshly prepared l-asparagine (40 mM) in Tris–HCl buffer (pH 8.6) and 100 µL of enzyme filtrate, incubated at 37 °C for 30 min and reaction was stopped by adding 100 µL of 1.5 M trichloroacetic acid (TCA). The reaction mixture was centrifuged at 10,000 rpm for 5 min at 4 °C to remove the precipitates. The ammonia released in the supernatant was determined using colorimetric technique by adding 200 µL of Nessler’s reagent into the sample containing 200 µL of supernatant and 1.6 mL distilled water. This mixture was vortexed and incubated at room temperature for 20 min. Absorbance was measured at 425 nm against the blanks that received TCA before the addition of enzyme. The ammonia liberated in the reaction was determined based on the standard curve obtained using ammonium sulfate. One unit (IU) of l-asparaginase activity was defined as the amount of the enzyme that liberates 1 µM of ammonia per min at 37 °C, using asparagine as substrate.

Extracellular protein content was determined using Lowry method (Lowry et al. 1951). Specific activity is expressed as unit enzyme activity per mg of protein.

## Results and discussion

### Isolation of fungal species

A total of 45 fungal species were isolated on the basis of zone formation from soil, wheat bran, rice husk, cotton seed oil cake and red gram feed. Among isolated fungi, 34 isolates were able to grow in secondary screening with MCD medium containing different nitrogen sources. Out of 45 fungal isolates, 27 were from soil implying that 60 % of isolated fungi were from soil samples, rest from agricultural residues. *Aspergillus* sp., *Penicillium* sp., *Trichophyton* sp. and *Onychocola* sp. were predominant fungi isolated from the soil samples. *Rhizopus* sp. and *Fusarium* sp. were isolated from agricultural residues. *Aspergillus* sp. was the most dominant species among fungi isolated from soil and agricultural residues. These results were comparable to previously reported studies (Qiao et al. 2008; Tančinová and Labuda 2009).

### Screening studies

**Fig. 1:**
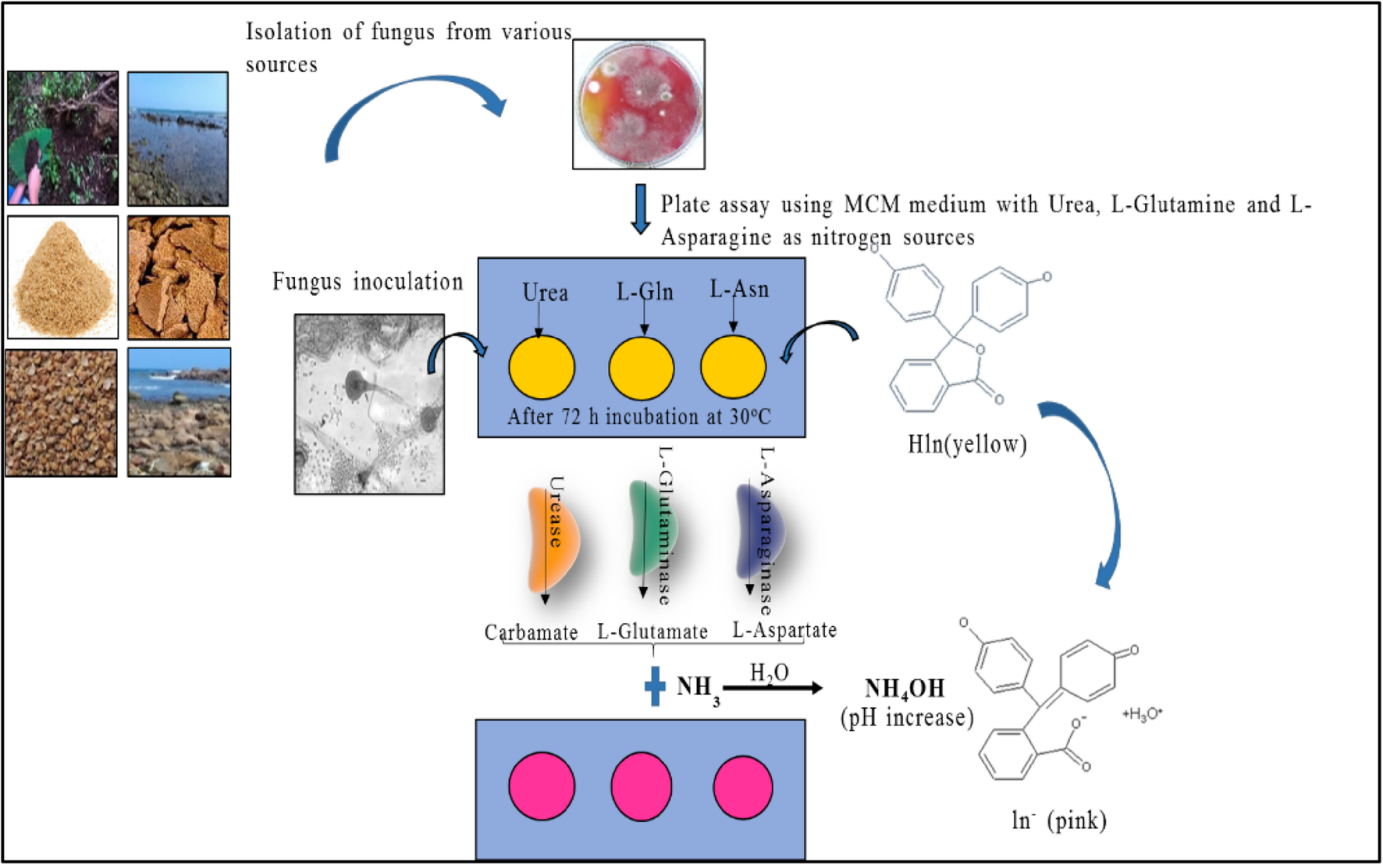
Amidohydrolases: urease, l-glutaminase, l-asparaginase convert urea, L-Gln, L-Asn, respectively, producing ammonia and acid resulting in increase in the pH with product formation. *Pink*-*coloured* zone around the colony indicates enzyme activity

Current study involved the screening of isolated fungi for the existence of three industrially important enzymes using phenol red and BTB dye. For screening of l-asparaginase, l-glutaminase and urease enzyme, MCD supplemented with l-Asn, l-Gln and urea, respectively, as the sole nitrogen sources are used. These amidohydrolases cleave amine groups and liberate aspartic acid and ammonia in case of l-asparaginase, glutamic acid and ammonia in case of l-glutaminase and carbonic acid and ammonia if urease is produced (as shown in Fig. 1). Ammonia liberated in the medium further reacts with water to produce NH_4_OH resulting in increase in the pH of the medium.

**Fig. 2:**
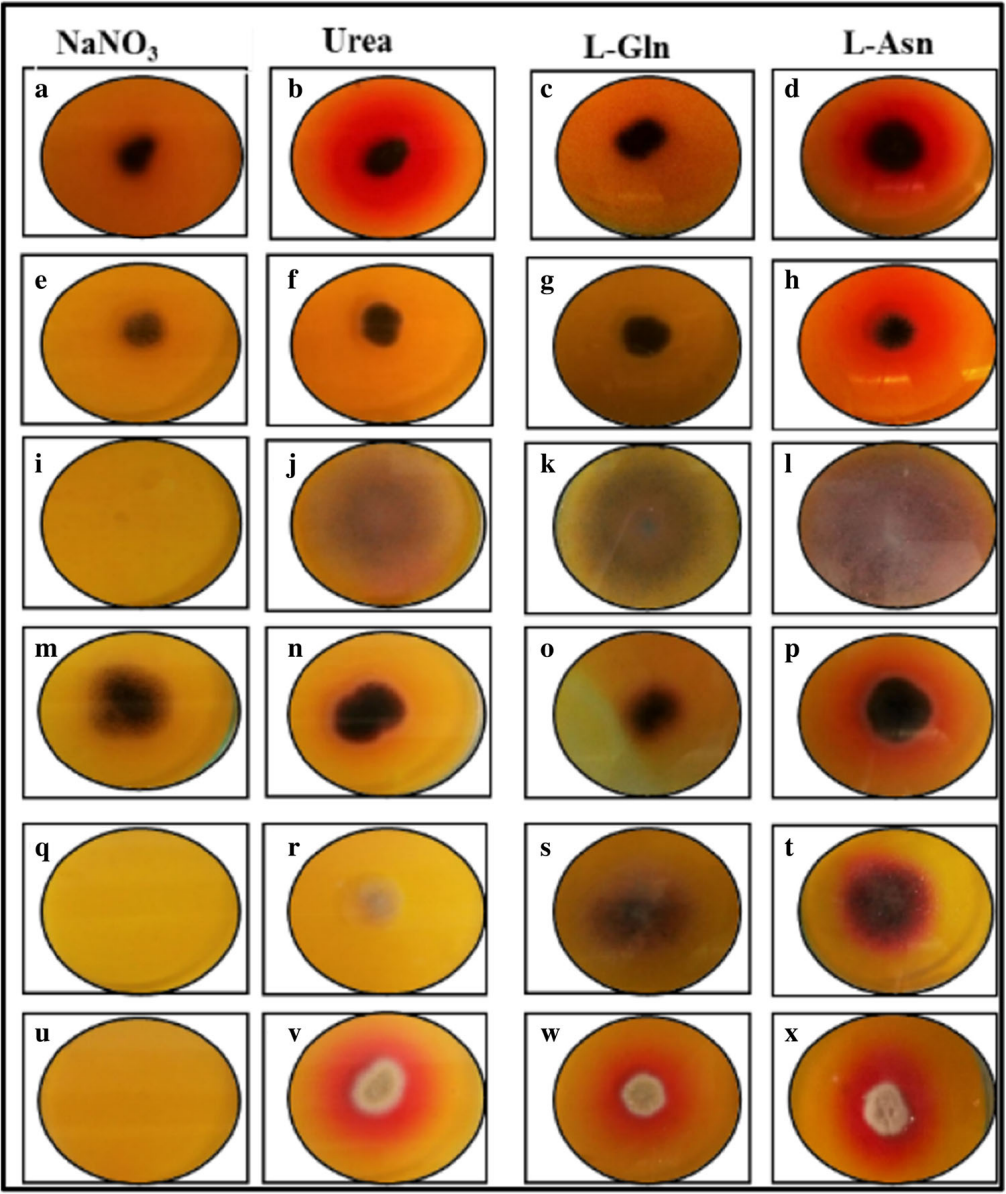
Assay for screening l-asparaginase-producing fungi amended with different substrates, on plate supplemented with phenol *red* dye. **a**–**d** S_3.4_ isolate grown on plates containing NaNO_3_, urea, l-Gln and l-Asn; **e**–**h** C_3_ isolate grown on plates containing NaNO_3_, urea, l-Gln and l-Asn; **i**–**l** W_5_ isolate grown on plates containing NaNO_3_, urea, l-Gln and l-Asn; **m**–**p** C_7_ isolate grown on plates containing NaNO_3_, urea, l-Gln and l-Asn; **q**–**t** W_3_ isolate grown on plates containing NaNO_3_, urea, l-Gln and l-Asn; **u**–**x** *Aspergillus terreus* MTCC 1782 strain grown on plates containing NaNO_3_, urea, l-Gln and l-Asn

**Fig. 3:**
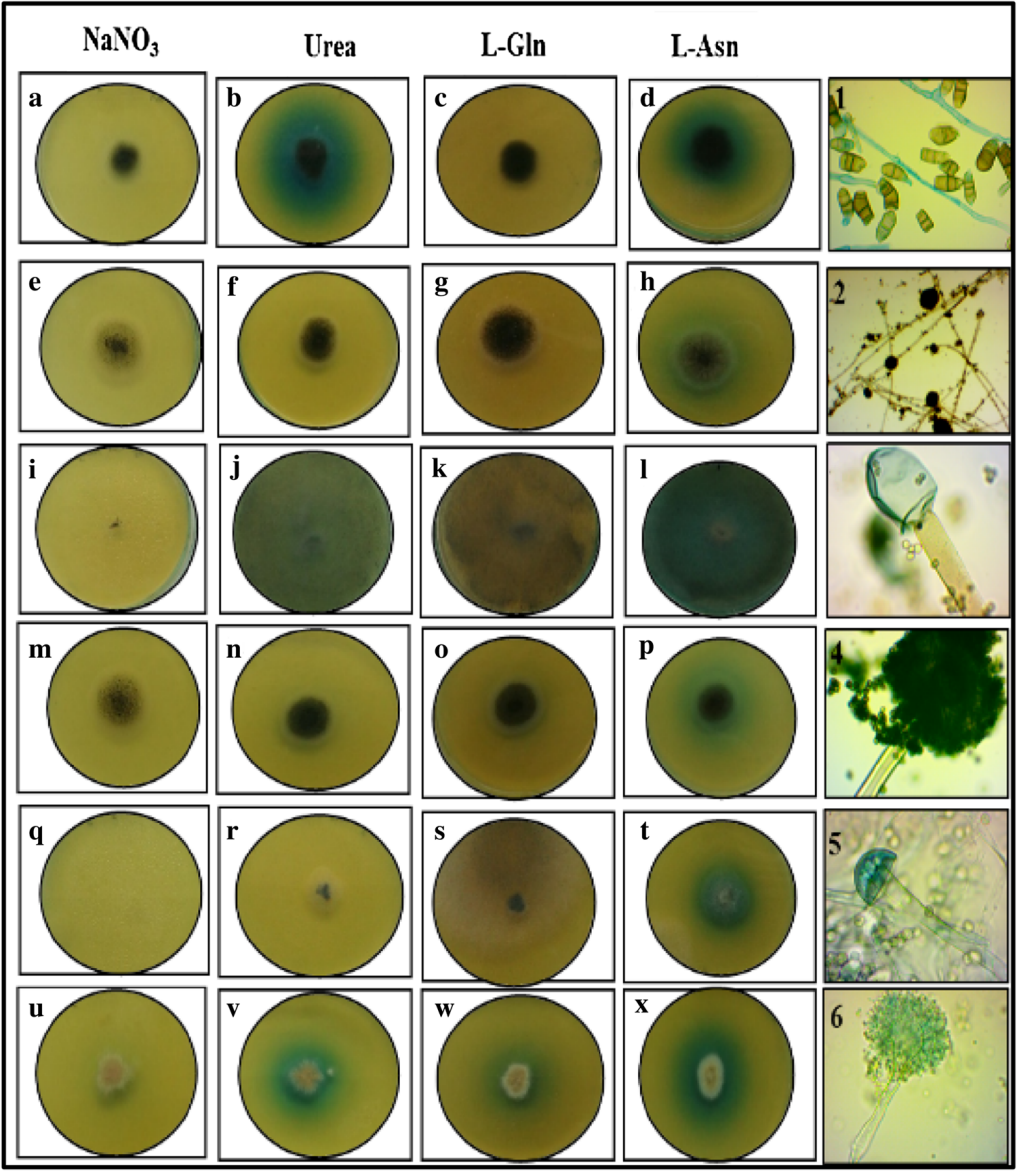
Assay for screening l-asparaginase-producing fungi amended with different substrates, on plate supplemented with BTB dye. **a**–**d** S_3.4_ isolate grown on plates containing NaNO_3_, urea, l-Gln and l-Asn; **e**–**h** C_3_ isolate grown on plates containing NaNO_3_, urea, l-Gln and l-Asn; **i**–**l** W_5_ isolate grown on plates containing NaNO_3_, urea, l-Gln and l-Asn; **m**–**p** C_7_ isolate grown on plates containing NaNO_3_, urea, l-Gln and l-Asn; **q**–**t** W_3_ isolate grown on plates containing NaNO_3_, urea, l-Gln and l-Asn **u**–**x**; *Aspergillus terreus* MTCC 1782 strain grown on plates containing NaNO_3_, urea, l-Gln and l-Asn; 1 S_3.4_, 2 C_3_, 3 W_5_, 4 C_7_, 5 W_3_, 6 MTCC 1782: microscopic images of isolates using light microscope ×40 magnification

Phenol red dye is yellow at acidic pH and turns pink at alkaline pH; presence of pink colour zone around the colonies on MCD plates with different nitrogen sources is due to the liberation of corresponding enzyme (Gulati et al. 1997). Thirty-four isolates showed pink zone around the colonies indicating increase in pH. In Fig. 2, last column shows presence of pink-coloured zone around fungal isolates S_3.4_, W_3_, W_5_, C_3_ and C_7_ in l-Asn plates indicating l-asparaginase activity. These isolates did not show any colour change in plates containing l-Gln connoting the absence of l-glutaminase. S_3.4_ and MTCC 1782 isolates produce the urease enzyme which is confirmed by the pink-coloured zone around the colony in plates with urea as nitrogen source. MTCC 1782 strain showed pink-coloured zone when grown on l-Asn, l-Gln and urea indicating that strain produces three enzymes. Strains W_3_, W_5_, C_3_ and C_7_ show pink colour zone only on l-asparagine plate, indicating strains are free of l-glutaminase and urease. To ensure reproducibility, all the isolates were screened with BTB as both the dyes are formulated for screening the hydrolysis of l-Gln, l-Asn and urea. Among phenol red and BTB, 0.007 % of BTB dye showed sharp colour contrast zone, ranging from yellow at acidic pH, green at neutral pH to blue at alkaline pH (Mahajan et al. 2013). MCD plates with different substrates supplemented with BTB dye is shown in Fig. 3. After 72 h of incubation, thirty-four isolates showed blue-coloured zone around the colonies indicating increase in pH.

In comparison with phenol red, hydrolysed and unhydrolyzed enzymes were clear and precise in MCD supplemented with BTB. Methyl red was incorporated as pH indicator in the recent study to screen l-asparaginase- and l-glutaminase-producing microorganism (Dhale Dhale and Mohan Kumari 2014). Enzyme activity is calculated semi-quantitatively by relative ratio of zone diameter to colony diameter. Level of enzyme production was indicated by zone index. The comparison of zone index values of isolates S_3.4_, W_3_, W_5_, C_3_, C_7_ and *Aspergillus* MTCC 1782 strain using phenol red and BTB dye is given in Table 2. Using this qualitative plate assay, rapid screening of the fungi for the synthesis of the enzyme by direct visualization and activity of the enzyme can be measured (Hankin and Anagnostakis 1975). Gulati et al. revealed that equivalent relation exists between zone index and enzyme activity measured from broth. In the current work, enzyme index varied from 0.8 to 4, which is in line with study conducted by Shrivastava et al. (2010). Enzyme index of C_7_ is 1.57 with colony diameter of 3.5 cm and zone diameter of 5.5 cm which is lower than that of MTCC 1782 strain with enzyme index of 2.40. Out of 34 isolated fungal species, only 4 isolates showed l-asparaginase free of l-glutaminase and urease as shown in Table 3. Isolated fungi (S_3.4_, W_3_, W_5_, C_3_ and C_7_) were cultured in PDA slants, later morphologically identified as *Curvularia* sp., *Rhizopus* sp. and *Aspergillus* sp., respectively (Ellis et al. 2007).

**Table 2:** l-Asparaginase enzyme index measurement using phenol red and bromothymol blue amended in MCD medium after 72 h incubation and species observed under light microscope

Isolate	Phenol red			Bromothymol blue			Species
	Colony diameter (cm)	Zone diameter (cm)	Zone index	Colony diameter (cm)	Zone diameter (cm)	Zone index	
S_3.4_	4.30	6.70	1.56	3.30	6.80	2.06	*Curvularia* sp.
W_3_	2.40	2.40	1.00	3.70	4.70	1.27	*Rhizopus* sp.
W_5_	8.80	8.80	1.00	2.20	2.60	1.18	*Rhizopus* sp.
C_3_	3.00	3.00	1.00	3.80	5.50	1.45	*Aspergillus* sp.
C_7_	3.00	4.60	1.53	3.50	5.50	1.57	*Aspergillus* sp.
MTCC 1782	2.50	6.00	2.40	2.50	6.00	2.40	*Aspergillus* sp.

**Table 3:** Fungal species screened for multi-enzyme production (amide)

S. no.	Isolation source	Isolate	Control (NaNO_3_)	Urea	L-Asn	L-Gln	l-Asparaginase enzyme index		
							Colony diameter (cm)	Zone diameter (cm)	Zone index
1	Soil from Vizag	V_1_	–	–	+	+	2.7	6.6	2.44
2		V_2_	–	+	+	+	2.2	5	2.27
3		V_3_	–	+	+	–	1	3	3.00
4		V_4_	–	+	+	+	3	6	2.00
5		V_5_	–	+	+	+	3.1	6	1.94
6		V_6_	–	+	+	–	1	3.1	3.10
7	Soil from Kanyakumari	K_1_	–	+	+	+	1	2.1	2.10
8		K_2_	–	–	+	+	2.4	5.8	2.42
9		K_3_	–	+	+	+	2.5	5.2	2.08
10		K_4_	–	–	+	+	2.1	4	1.90
11		K_5_	–	–	+	+	1.7	4.7	2.76
12		K_6_	–	–	+	+	2.5	6.8	2.72
13		K_7_	–	+	+	+	0.7	1.8	2.57
14		K_8_	–	–	+	+	1.25	4.4	3.52
15	Soil from Western Ghats	S_1.1_	–	+	+	+	2	6.5	3.25
16		S_1.4_	–	+	+	+	2.1	3.9	1.86
17		S_2.1_	–	+	+	+	3.2	8.5	2.66
18		S_3.4_	–	+	+	–	3.3	6.8	2.06
19		S_4.1_	–	+	+	+	2	8.5	4.25
20	Red gram husk	P_2_	–	+	+	+	3.5	5.5	1.57
21		P_3_	–	+	+	+	2.5	3	1.20
22	Rice husk	R_1_	–	+	+	+	2.6	6	2.31
23		R_3_	–	+	+	+	3.7	6.3	1.70
24	Wheat bran	W_1_	–	+	+	–	6	6.5	1.08
25		W_2_	–	+	+	+	4	7	1.75
26		W_3_	–	–	+	–	3.7	4**.7**	1.27
27		W_4_	–	+	+	+	2	4	2.00
28		W_5_	–	–	+	–	2.2	2.6	1.18
29	Cotton seed oil cake	C_1_	–	+	+	+	2.5	6	2.40
30		C_3_	–	–	+	–	3.8	5.5	1.45
31		C_4_	–	+	+	–	7.5	7	0.93
32		C_5_	–	+	+	–	7.5	7	0.93
33		C_6_	–	+	+	+	8.5	7	0.82
34		C_7_	–	–	+	–	3.5	5.5	1.57
35		MTCC 1782	–	+	+	+	2.5	6.0	2.4

V (1–6) soil from Visakhapatnam (Vizag), K (1–8) soil from Kanyakumari, S (1.1, 1.4, 2.1, 3.4 and 4.1) soil from Western Ghats, P (2–4) red gram husk, R (1 and 3) rice husk, W (1–5) wheat bran, C (1, 3, 4, 5, 6 and 7) cotton seed oil cake

**Fig. 4:**
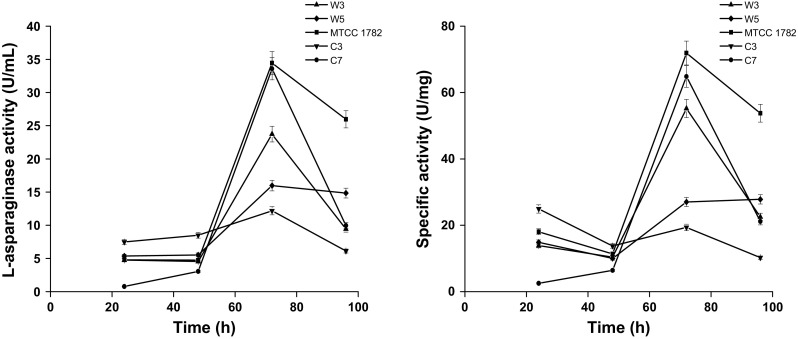
l-Asparaginase activity and specific activity of isolated strains

The l-asparaginase activity of the four isolated strains with no glutaminase and urease activity is measured in liquid broth studies along with MTCC 1782 (shown in Fig. 4). MTCC 1782 strain is found to have the highest activity at 72 h with l-asparaginase activity of 34.45 U/mL and specific activity of 71.92 U/mg. Reported activity for optimized *Aspergillus terreus* MTCC 1782 was 40.186 IU/mL (Baskar and Renganathan 2012). Among the four isolated strains C_7_ has highest activity of 33.59 U/mL and specific activity of 64.85 U/mg. Hence, medium has to be developed and optimized for l-asparaginase production from C_7_ to enhance l-asparaginase activity. All the strains exhibit the maximum activity at 72 h.

### Effect of carbon and nitrogen sources

**Fig. 5:**
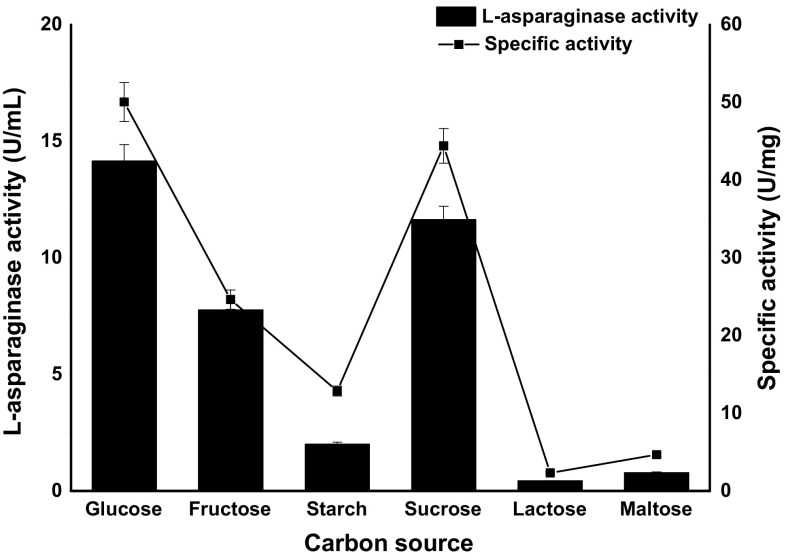
l-Asparaginase activity and specific activity of C_7_ strain with different carbon sources

**Fig. 6:**
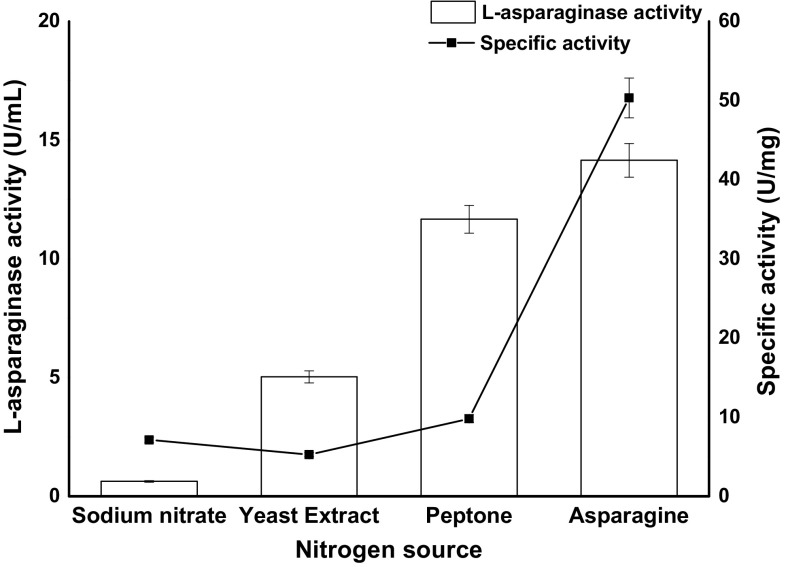
l-Asparaginase activity and specific activity of C_7_ strain with different nitrogen sources

**Fig. 7:**
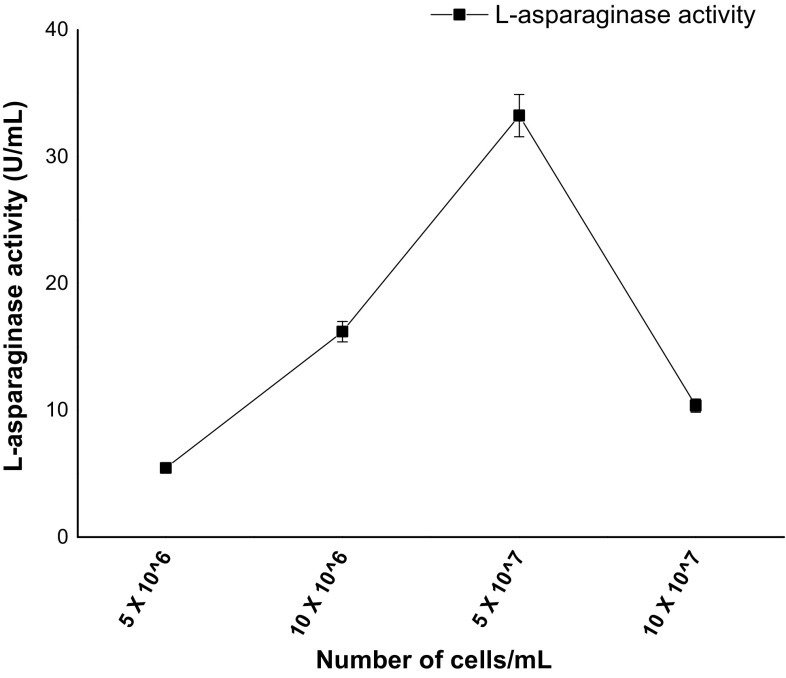
Effect of inoculum on l-asparaginase activity using C_7_ measured after 72-h incubation

Six different carbon sources (fructose, glucose, maltose, sucrose, lactose and starch) were evaluated for the l-asparaginase production by C_7_ which is free of glutaminase and urease. Batch cultivation of C_7_ in MCD medium using different carbon sources revealed distinctive variations on l-asparaginase production and specific activity (Fig. 5). In comparison to other carbon sources, C_7_ produced maximum asparaginase (16.2 U/mL) when glucose is used as a carbon source; lactose, maltose and starch were the poorest carbon sources. Sucrose and fructose also supported l-asparaginase production to a significant degree but glucose acted as good inducer and primary source of carbon for biosynthesis of l-asparaginase using C_7_. Several reports suggest that glucose serves as a best carbon source for l-asparaginase production and a similar effect was observed for l-asparaginase production using *Aspergillus* and *Fusarium* strains (Baskar and Renganathan 2012; Hosamani and Kaliwal 2011). Effect of nitrogen compounds on l-asparaginase by C_7_ was studied by supplementing nitrogen sources (asparagine, yeast extract, peptone and sodium nitrate) to MCD medium. C_7_ amended with asparagine favoured maximum enzyme production indicating l-asparagine itself acts as a nitrogen source and influence l-asparaginase production (Fig. 6). Peptone also supported the production of l-asparaginase to a substantial quantity. A considerable decrease in enzyme activity was observed when C_7_ was amended with yeast extract. Lower enzyme activity was detected in the media supplemented with sodium nitrate; on the contrary, a study in which *Fusarium oxysporum* has shown higher enzyme production with sodium nitrate as a nitrogen source (Tippani and Sivadevuni 2012). Effect of inoculum volume on l-asparaginase production by C_7_ is shown in Fig. 7. At low inoculum concentration, the l-asparaginase production was less, and the enzyme activity increased with increase in inoculum volume. At inoculum volume of 5 × 10^7^ cells/mL, enzyme activity is 33.59 U/mL. With further increase in inoculum concentration, the biosynthetic activity decreased due to nutrient depletion.

Most of the l-asparaginase purified from various sources such as chillies and *E. coli* shows specificity towards both L-Gln and urea. Several studies reveal that specificity of l-asparaginase is important in selective depletion of asparagine-dependent tumour cells (Hill et al. 1967; Durden and Distasio 1981; Distasio et al. 1982). To reduce the toxic effects associated with bacterial l-asparaginase, fungi is preferred as being eukaryotic and evolutionarily closer to human. It can minimize the chances of immunological reactions (Shrivastava et al. 2012). Several fungal endophytes were isolated from various sources and tested for their ability to synthesize l-glutaminase-free l-asparaginase. l-Glutaminase-free l-asparaginase produced by endophytic fungi from seaweed was isolated, later identified as *Fusarium, Alternaria* sp.*, Aspergillus* sp. and *Colletotrichum* sp. (Thangavel et al. 2013). *Alternaria* sp. endophytic fungi isolated from the leaf of *Withania somnifera* of Western Ghats is reported to show maximum l-asparaginase activity that is free of l-glutaminase (Nagarajan et al. 2014). In the current study, 45 fungi isolates were subjected to screening, with a view to assess the isolates for their ability to utilize different substrates as a nitrogen source. Twenty fungi isolates have shown the presence of urease, l-glutaminase and l-asparaginase enzyme. Four isolates have shown the presence of l-asparaginase free of urease and l-glutaminase, and six isolates presented l-asparaginase free of l-glutaminase with presence of urease using plate assay. Fungal isolates were selected on the basis of zone formation around the colonies, when grown on MCD with phenol red or BTB as a pH indicator. The change in colour stated the accumulation of ammonia which resulted due to the hydrolysis of amidohydrolase (Singh and Srivastava 2012). Fungi secrete numerous enzymes into the medium and regulation of other contaminating enzymes would make it possibly the preferred drug in the treatment of cancer.

Current preparation of asparaginase used in treatment protocols are *E*. *coli* asparaginase, its PEGylated form and *Erwinia* asparaginase; several studies on different other sources of asparaginase have yielded encouraging outcomes. Further studies and regulatory supports will allow the introduction of new asparaginase drugs with potential benefits to patients. Fungal strain, namely C_7_, which is an l-asparaginase (free of l-glutaminase and urease)-producing strain has shown highest enzyme activity of 33.59 U/mL with carbon source as glucose; asparagine as nitrogen source at inoculum volume of 5 × 10^7^ cells/mL is to be considered for further study on purification and characterization of l-asparaginase enzyme.

## Abbreviations

ALL: Acute lymphoblastic leukaemia
BTB: Bromothymol blue
L-Asn: l-Asparagine
L-Gln: l-Glutamine
MCD: Modified Czapek–Dox
